# CNTN5 Promotes Invasion and Metastasis in Non‐Small Cell Lung Cancer by Regulating YAP1 Nuclear Translocation

**DOI:** 10.1002/jgm.70112

**Published:** 2026-09-21

**Authors:** Jiarui Wang, Mengtao Wang, Yanze Yin, Ao Zeng, Keyi Chen, Zhilong Xu, Jie Dai

**Affiliations:** ^1^ Department of Thoracic Surgery, Shanghai Pulmonary Hospital, School of Medicine Tongji University Shanghai China

**Keywords:** CNTN5, invasion, metastasis, non‐small cell lung cancer, prognosis, YAP1

## Abstract

**Background:**

Non‐small cell lung cancer (NSCLC) is a leading cause of cancer‐related death worldwide, largely owing to its high metastatic potential. Contactin‐5 (CNTN5), a glycosylphosphatidylinositol‐anchored membrane protein, has been implicated in tumor‐related processes; however, its role in NSCLC progression remains unclear. This study aimed to investigate the clinical relevance, biological function, and underlying mechanism of CNTN5 in NSCLC.

**Methods:**

CNTN5 expression was evaluated using public databases and clinical NSCLC specimens. Gain‐ and loss‐of‐function assays were performed in NSCLC cell lines. Cell proliferation, migration, and invasion were assessed using CCK‐8, wound healing, and Transwell assays, respectively. An experimental lung metastasis model was used to determine metastatic capacity in vivo. RNA sequencing and enrichment analysis were performed to explore the molecular mechanisms involved.

**Results:**

CNTN5 was upregulated in NSCLC tissues and was associated with poor prognosis. CNTN5 overexpression significantly enhanced NSCLC cell migration and invasion, while increased CNTN5 expression promoted lung metastatic colonization in vivo. Transcriptomic and protein analyses indicated that CNTN5 was associated with alterations in Hippo signaling pathway. In addition, CNTN5 increased YAP1 expression and promoted its nuclear translocation, while YAP1 silencing partially reversed the pro‐migratory and pro‐invasive effects induced by CNTN5. Co‐immunoprecipitation analyses further supported an interaction between CNTN5 and PTPN13, accompanied by enhanced nuclear localization of YAP1.

**Conclusions:**

CNTN5 promotes NSCLC progression, particularly invasion and metastasis, and is associated with unfavorable clinical outcomes. Mechanistically, CNTN5 enhances YAP1 nuclear translocation and activates YAP1‐associated transcriptional programs, highlighting its potential as a prognostic biomarker and therapeutic target in NSCLC.

## Graphical abstract

Contactin‐5 (CNTN5) is upregulated in non‐small cell lung cancer (NSCLC) and associated with metastasis and poor prognosis. Mechanistically, CNTN5 interacts with PTPN13 to promote YAP1 nuclear translocation and transcriptional activation, enhancing NSCLC cell migration and invasion in vitro and metastasis in vivo.

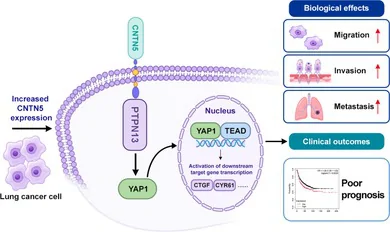

**Abbreviations**
CCK‐8: Cell Counting Kit‐8
CNTN5: Contactin‐5
Co‐IP: Co‐immunoprecipitation
DEGs: Differentially expressed genes
FP: First progression
GPI: Glycosylphosphatidylinositol
GSEA: Gene set enrichment analysis
H&E: Hematoxylin and eosin
HPA: Human Protein Atlas
IHC: Immunohistochemistry
KEGG: Kyoto Encyclopedia of Genes and Genomes
LUAD: Lung adenocarcinoma
LUSC: Lung squamous cell carcinoma
NSCLC: Non‐small cell lung cancer
OS: Overall survival
PTPN13: Protein tyrosine phosphatase nonreceptor type 13
RT‐qPCR: Quantitative reverse transcription polymerase chain reaction
siRNA: Small interfering RNA
TCGA: The Cancer Genome Atlas
YAP1: Yes‐associated protein 1

## 1 Introduction

Non‐small cell lung cancer (NSCLC) is one of the most prevalent malignancies worldwide, with the highest incidence and mortality rates, posing a serious threat to human health [1]. Although advances in targeted therapy, immunotherapy, and multimodal treatment have improved clinical outcomes, metastatic progression continues to be the principal cause of treatment failure and death in patients with NSCLC [2, 3]. Therefore, there is an urgent need to elucidate metastasis‐associated molecular determinants for risk stratification and therapeutic targeting [4].

Contactin‐5 (CNTN5) is a glycosylphosphatidylinositol (GPI)‐anchored neuronal membrane protein [5]. It plays an important role in nervous system development and neuropsychiatric disorders [6, 7, 8, 9]. Emerging evidence suggests that CNTN5 may also participate in physiological and pathological processes beyond the nervous system, including cardiovascular and immune‐inflammatory diseases [10, 11, 12]. However, the role of CNTN5 in cancer development and progression remains largely unknown [13]. Although previous studies have shown that the expression of CNTN5 is associated with the prognosis of head and neck squamous cell carcinoma and the occurrence and development of thyroid cancer, direct experimental evidence supporting its biological function and molecular mechanisms in cancer progression is still lacking [14, 15]. In particular, whether CNTN5 contributes to NSCLC invasion and metastasis and the signaling pathways underlying its effects have not yet been systematically elucidated.

In this study, we systematically investigated the biological role of CNTN5 in NSCLC progression and explored its underlying molecular mechanisms. By integrating in vitro and in vivo assays, transcriptomic sequencing, and protein interaction analyses, we demonstrated that CNTN5 enhances the invasive and migratory capacities of tumor cells and identified the Hippo signaling pathway as a critical downstream mediator of tumor progression. This study aimed to clarify the association between CNTN5 expression and the clinical characteristics and prognosis of NSCLC patients and to further elucidate the functional role and potential mechanisms of CNTN5 in NSCLC invasion and metastasis.

## 2 Materials and Methods

### 2.1 Clinical Data and Sample Collection

Clinical specimens used for RNA sequencing and immunohistochemical analyses were obtained from Shanghai Pulmonary Hospital, and the corresponding clinicopathological data were retrieved from medical records, as previously described [16]. Approval for the collection and utilization of clinical samples was granted by the Ethics Committee of Shanghai Pulmonary Hospital (Ethical approval number: K23–228). Informed consent was obtained from all subjects and/or their legal guardian(s). All procedures involving human specimens were conducted in accordance with the Declaration of Helsinki.

### 2.2 RNA Sequencing

A total of 40 fresh‐frozen NSCLC tissue samples from our cohort were subjected to RNA sequencing analysis. Total RNA was extracted using standard procedures and assessed for quality. Qualified RNA samples were quantified, amplified, fragmented, and used for library construction followed by high‐throughput sequencing. In addition, total RNA was isolated from CNTN5‐overexpressing H1299 cells and corresponding control cells. RNA sequencing of both tissue and cell samples was performed by Novobio (Shanghai, China). Differentially expressed genes (DEGs) were identified based on |log2 (fold change)| and adjusted *p*‐values. Functional enrichment analyses of DEGs were conducted using gene set enrichment analysis (GSEA), Kyoto Encyclopedia of Genes and Genomes (KEGG) pathway analysis, and protein–protein interaction (PPI) network analysis.

### 2.3 Immunohistochemistry (IHC) Analysis

Paraffin‐embedded NSCLC tissue sections were deparaffinized and rehydrated, followed by blocking of endogenous peroxidase activity with 3% H_2_O_2_. Antigen retrieval was performed by microwave heating in citrate buffer. The sections were incubated with anti‐CNTN5 primary antibody at 4°C overnight, followed by incubation with HRP‐conjugated secondary antibody. CNTN5 expression was visualized using DAB staining, and nuclei were counterstained with hematoxylin. Staining intensity was quantified using ImageJ software.

### 2.4 Quantitative Reverse Transcription PCR (RT‐qPCR)

Total RNA was extracted from cell lines using TRIzol reagent (Servicebio Technology, Wuhan, China). Reverse transcription was performed using the HiScript III RT SuperMix for qPCR kit (Vazyme, Shanghai, China). RT‐qPCR was conducted using SYBR Green Pro Taq HS Premix (AG, Shanghai, China) according to the manufacturer's instructions. Relative gene expression levels were calculated using the 2 − ΔΔCt method, and values were normalized against β‐actin. The primer sequences used are listed in Table S1.

### 2.5 Cell Culture

The normal human bronchial epithelial cell line BEAS‐2B and NSCLC cell lines PC‐9, A549, H1975, H520, and H1299 were originally from the Cell Bank of the Chinese Academy of Sciences (Shanghai, China). H1975 and H1299 cells were cultured in RPMI‐1640 medium (Servicebio Technology, Wuhan, China). BEAS‐2B, PC‐9, A549, H520, and Lewis lung carcinoma cells (LLC) were cultured in Dulbecco's modified eagle medium (DMEM) (Servicebio Technology, Wuhan, China). All media were supplemented with 10% fetal bovine serum (FBS; Gibco, USA). Cells were cultured at 37°C with 5% CO_2_ in a humidified environment.

### 2.6 Cell Transfection

CNTN5‐overexpressing, Cntn5‐overexpressing, and control lentiviruses were purchased from Tsingke (Shanghai, China). The H1975, H1299, and Lewis cells were transduced with lentiviruses and selected with 2 μg/mL of puromycin for 5 days to establish CNTN5 or Cntn5 overexpression (OE) cells.

The siRNAs against CNTN5, PTPN13, and YAP1 were purchased from Jieli (Shanghai, China). H1975 and H1299 cells were transfected with siRNA using the Lipofectamine 2000 (Invitrogen, USA) according to the manufacturer's instructions. The cells were harvested at 48 h after transfection for use. A nonspecific scramble siRNA sequence was used as negative control (NC). The sequences of siRNAs are summarized in Table 1.

**TABLE 1:** Sequences of siRNAs used in this study.

siRNA name	Sense strand (5′ → 3′)	Antisense strand (5′ → 3′)
si‐CNTN5	GAUUAUCGCUACAGUUUGA (dT)(dT)	UCAAACUGUAGCGAUAAUC (dT)(dT)
si‐YAP1	GGUCAGAGAUACUUCUUAA (dT)(dT)	UUAAGAAGUAUCUCUGACC (dT)(dT)
si‐PTPN13	CUAGUUCGAUGGAUAAGUA (dT)(dT)	UACUUAUCCAUCGAACUAG (dT)(dT)

### 2.7 Protein Extraction and Western Blotting

To extract proteins, cell samples were lysed in RIPA lysis buffer (Servicebio Technology, Wuhan, China) containing protease inhibitor (Beyotime, Shanghai, China). Protein concentrations were measured using the BCA Protein Kit (Servicebio Technology, Wuhan, China). Proteins were separated by 4%–12% SDS‐PAGE gel electrophoresis and transferred to PVDF membranes (Millipore). The membranes were blocked with 5% skimmed milk for 2 h at room temperature, followed by an overnight incubation with specific primary antibodies at 4 C. The membranes were incubated with HRP‐conjugated secondary antibody for 1.5 h at room temperature, and finally, protein bands were visualized using an ECL kit (Servicebio Technology, Wuhan, China). Co‐immunoprecipitation (Co‐IP) assays were performed using Protein A/G Magnetic Beads (Vazyme, Nanjing, China) according to the manufacturer's instructions. The immunoprecipitated proteins were analyzed by western blotting.

### 2.8 Wound Healing Assay

Cells were cultured in six‐well plates and cultured until 100% confluence. A straight cell‐free wound area was created using a sterile 100‐μL pipette tip. The cells were gently washed with PBS to remove detached cells and then cultured in serum‐free medium. Images of the wound area were captured at 0 and 24 h. Wound healing was quantified using ImageJ software.

### 2.9 Transwell Assay

The 24‐well plates containing Transwell chambers (8‐μm pore membranes; Corning Inc., NY, USA) were used to perform migration and invasion assays. Briefly, 1 × 10^5^ cells were suspended in serum‐free medium and seeded into each upper Transwell chamber (Corning Inc., NY, USA) with or without Matrigel (Corning Inc., NY, USA), and 600 μL of medium containing 20% FBS was added to the lower chambers. After 24 h of incubation in the cell culture incubator, cells were fixed with paraformaldehyde for 30 min and stained with 0.5% crystal violet for 30 min. Cell counting was performed using a microscope and analyzed using ImageJ software.

### 2.10 Cell Counting Kit‐8 (CCK‐8) Assay

The Cell Counting Kit‐8 (Beyotime, Shanghai, China) was used to examine cell proliferation. 1 × 10^3^ cells were seeded into 96‐well plates and cultured for 0, 24, 48, 72, and 96 h. A mixture of CCK‐8 (10 μL) and complete medium (100 μL) was added to each well and incubated at 37°C for 1 h. The cell absorbance was then measured at 450 nm using a spectrophotometer.

### 2.11 Immunofluorescence Staining

The cells were fixed with 4% paraformaldehyde, permeabilized with Triton X‐100, blocked with normal goat serum at 37°C for 1 h, and incubated with primary antibody (CNTN5: 1:200, Themofisher, USA, YAP: 1:100, CST) overnight at 4°C. Next, they were incubated with fluorescein isothiocyanate‐conjugated (FITC) or tetramethylrhodamine isothiocyanate‐conjugated (TRITC) secondary antibody at 37°C for 2 h. Cell nuclei were stained with 4,6‐diamidino‐2‐phenylindole (DAPI). Cell images were acquired and analyzed using an inverted Nikon TE300 microscope (Nikon Co., Ltd.).

### 2.12 Animal Experiments

Male BALB/c nude mice (8 weeks old) were purchased from Shanghai Laboratory Animal Center (Shanghai, China). All animal experiments were approved by the Committee on the Ethics of Animal Experiments of Shanghai Pulmonary Hospital (ethical approval number: K21‐097Y) and were conducted in accordance with institutional guidelines. H1975 or Lewis cells were harvested, counted, and resuspended in 0.2 mL of sterile phosphate‐buffered saline (PBS). Each mouse was injected with 2 × 10^6^ tumor cells via the tail vein to establish an experimental lung metastasis model. Fourteen days after injection, the mice were humanely euthanized and subjected to necropsy. Lung tissues were collected to evaluate tumor growth and metastatic burden. The excised tissues were fixed in 4% paraformaldehyde (Servicebio Technology, Wuhan, China), embedded in paraffin, sectioned, and stained with hematoxylin and eosin (H&E). Tumor nodules in the lung sections were examined under a light microscope.

### 2.13 Statistical Analysis

Statistical analyses were performed using GraphPad Prism (version 10.0) and R software (version 4.4.4). Data are expressed as the mean ± standard deviation (SD). No data transformation, normalization, or outlier removal was performed unless otherwise specified. For in vitro experiments, data were obtained from at least three independent experiments (*n* ≥ 3). Sample sizes for other experiments are specified in the corresponding experimental sections and figure legends. Differences between two independent groups were assessed using the two‐tailed unpaired Student's *t*‐test, whereas paired data were analyzed using the two‐tailed paired Student's *t*‐test. CCK‐8 data and body weight data from animal experiments were analyzed using two‐way ANOVA. The alpha value was set at 0.05, and *p* < 0.05 was considered statistically significant.

## 3 Results

### 3.1 CNTN5 Is Upregulated in NSCLC and Associated With Poor Prognosis

**FIGURE 1:**
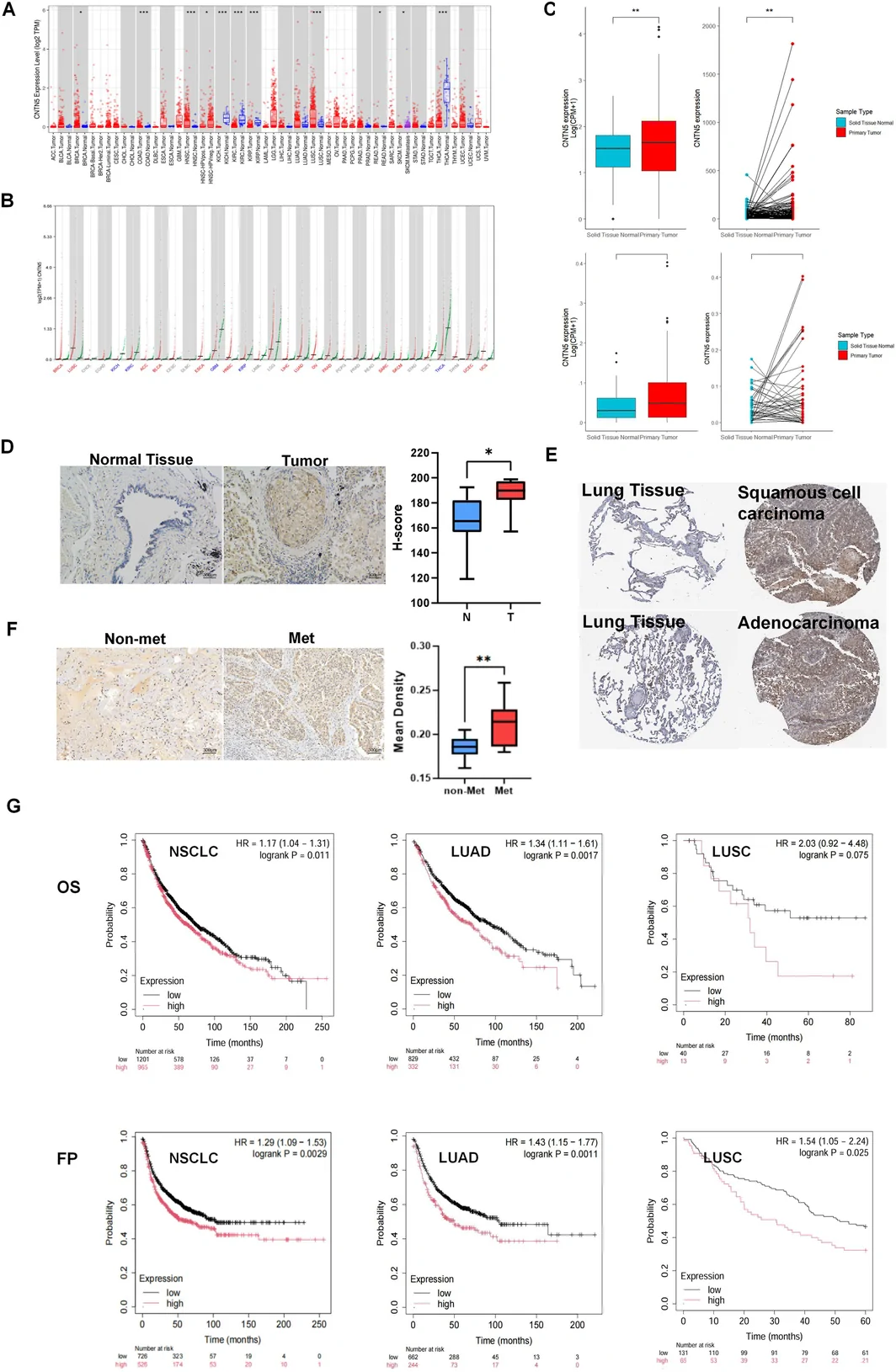
CNTN5 is upregulated in NSCLC and is associated with poor prognosis. (A) Pan‐cancer analysis of CNTN5 expression using TIMER. (B) Pan‐cancer analysis of CNTN5 mRNA expression using GEPIA. (C) CNTN5 mRNA expression in LUAD and LUSC tissues in the TCGA database. (D) CNTN5 protein expression in NSCLC and adjacent normal tissues assessed by IHC (n = 13). (E) Representative IHC images of CNTN5 expression in normal lung and lung cancer tissues obtained from the Human Protein Atlas. Image credit: Human Protein Atlas. The images are available at https://v25.proteinatlas.org/ENSG00000149972‐CNTN5/tissue/lung#img and https://v25.proteinatlas.org/ENSG00000149972‐CNTN5/cancer/lung+cancer#LUAD_TCGA. HPA content is licensed under the Creative Commons Attribution 4.0 International License. (F) CNTN5 protein expression in tumor tissues from patients with or without metastasis (*n* = 14 with metastasis and *n* = 8 without metastasis). (G) Survival curves of OS and FP in NSCLC. NSCLC, non‐small cell lung cancer; LUAD, lung adenocarcinoma; LUSC, lung squamous cell carcinoma; OS, overall survival; FP, first progression; CNTN5, contactin 5; scale bars = 300 μm. Statistical comparisons were performed using the two‐tailed paired Student's *t*‐test for paired samples and the two‐tailed unpaired Student's *t*‐test for independent samples. *p < 0.05, **p < 0.01, ***p < 0.001.

To investigate the expression pattern and clinical significance of CNTN5 in NSCLC, we first performed bioinformatics analyses. TIMER database analysis revealed that CNTN5 expression was upregulated to varying degrees in multiple tumor types, compared with corresponding normal tissues [17] (Figure 1A). Similarly, the GEPIA online database showed that CNTN5 was significantly upregulated in 14 cancer types versus normal tissues, including lung squamous cell carcinoma (LUSC), lung adenocarcinoma (LUAD), breast cancer, colorectal cancer, and head and neck squamous cell carcinoma [18] (Figure 1B).

To further clarify CNTN5 expression in NSCLC, we analyzed data from The Cancer Genome Atlas (TCGA). The results demonstrated that CNTN5 mRNA expression was significantly higher in tumor tissues than in adjacent normal lung tissues in both unpaired and paired analyses (*p* < 0.01, Figure 1C). Consistently, RNA‐seq data from our own cohort confirmed the upregulation of CNTN5 in NSCLC tissues (*p* < 0.05, Figure 1C). At the protein level, analyses of the Human Protein Atlas (HPA, proteinatlas.org) database and immunohistochemical (IHC) staining of clinical specimens showed that CNTN5 protein expression were increased in NSCLC tissues compared with normal lung tissues [19] (Figure 1D,E).

We further explored the association between CNTN5 expression and clinicopathological characteristics of NSCLC patients. IHC scoring of clinical samples demonstrated CNTN5 was highly expressed in tumor tissues from patients with metastasis (*p* < 0.05, Figure 1F). Kaplan–Meier survival analysis was performed to assess the prognostic value of CNTN5 in NSCLC. The results showed that high CNTN5 expression in tumor tissues was significantly associated with shorter overall survival (OS) and first progression (FP) in NSCLC patients (*p* < 0.001, Figure 1G). Collectively, these findings demonstrated that CNTN5 is highly expressed in NSCLC and closely associated with tumor aggressiveness and poor patient prognosis, suggesting that CNTN5 may serve as a potential prognostic biomarker in NSCLC.

### 3.2 CNTN5 Promotes Migration and Invasion of NSCLC Cells

**FIGURE 2:**
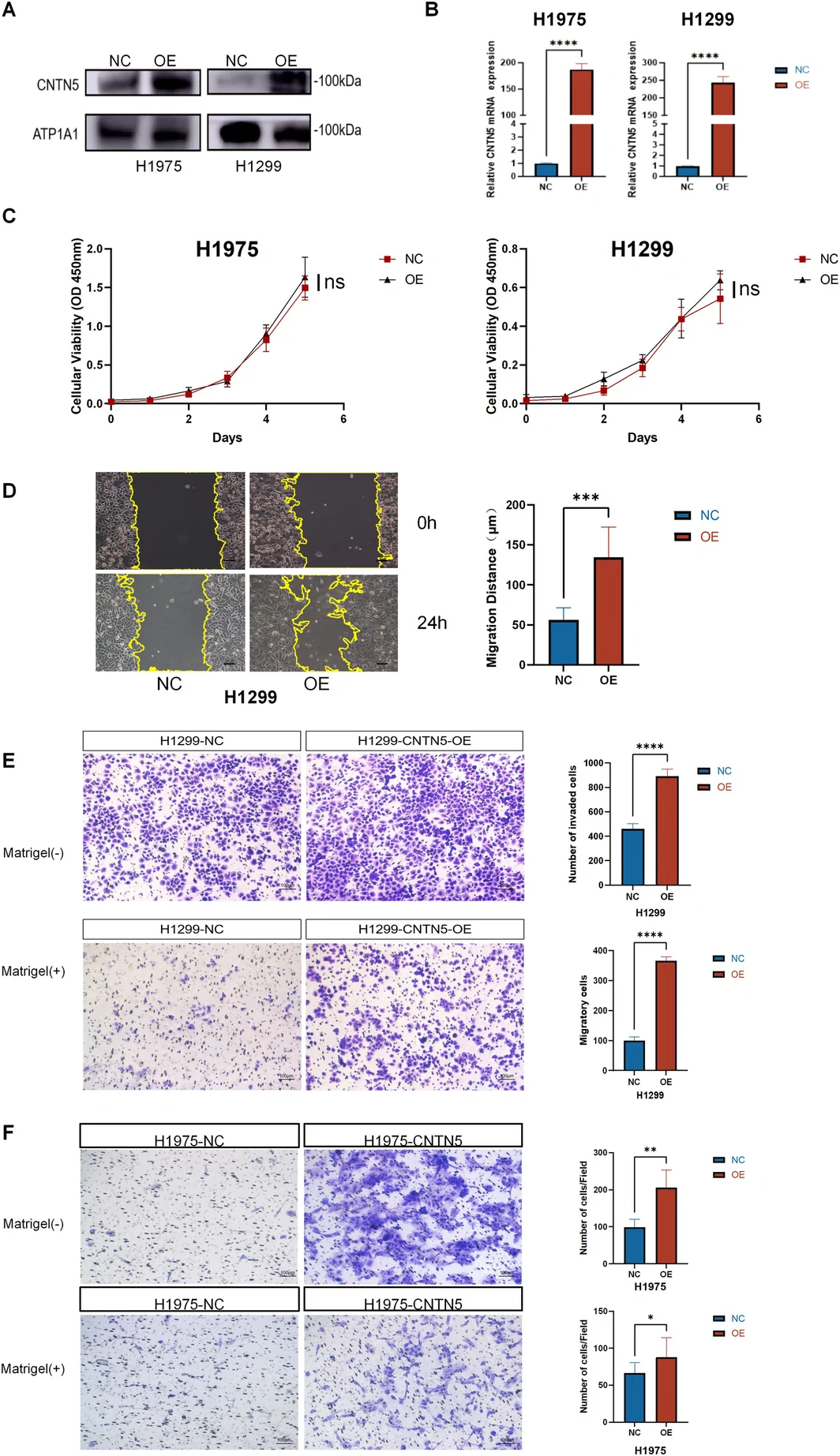
CNTN5 promoted migration and invasion of NSCLC cells. (A, B) CNTN5 overexpression in H1975 and H1299 cells following lentiviral transduction was confirmed by western blotting (A) and RT‐qPCR (B). (C) CCK‐8 assay showed that CNTN5 overexpression did not significantly affect the proliferation of H1975 and H1299 cells. (D) Cell migration in H1975 cells was assessed using a wound healing assay. (E, F) Cell migration and invasion in H1975 and H1299 cells were assessed using Transwell assays. Data are presented as the mean ± SD from at least three independent experiments (*n* ≥ 3). Comparisons were performed using the two‐tailed unpaired Student's *t*‐test. CCK‐8 data were analyzed using two‐way ANOVA. Scale bar = 100 μm. *p < 0.05, **p < 0.01, ***p < 0.001, ****p < 0.0001.

To further validate CNTN5 expression in NSCLC cells, we examined CNTN5 expression in normal lung epithelial cells and NSCLC cell lines. CNTN5 mRNA and protein levels were higher in NSCLC cell lines than in normal lung epithelial cells, supporting the use of these cell models for subsequent functional assays (Figure S1). To elucidate the biological function of CNTN5 in NSCLC, we established CNTN5 overexpressing cell lines in H1299 and H1975 cells using a lentiviral system. Overexpression efficiency was confirmed by western blotting and RT‐qPCR (Figure 2A,B).

Cell proliferation was evaluated using the CCK‐8 assay. The results showed that CNTN5 overexpression did not significantly affect the proliferative capacity of NSCLC cells compared with control cells (Figure 2C). Flow cytometric analyses further demonstrated that CNTN5 overexpression had no significant impact on apoptosis or cell cycle distribution (Figure S2).

In contrast, Transwell assays revealed that CNTN5 overexpression significantly enhanced the migratory and invasive abilities of NSCLC cells (Figure 2E,F). Consistently, wound healing assays showed that CNTN5‐OE cells exhibited a faster migration rate than control cells (Figures 2D and S3A). To further validate these findings, CNTN5 expression was silenced using siRNA, which resulted in a marked suppression of cell migration and invasion (Figure S3B). These results indicate that CNTN5 primarily promotes NSCLC cell migration and invasion rather than affecting cell proliferation or survival.

### 3.3 CNTN5 Promotes Tumor Metastasis In Vivo

**FIGURE 3:**
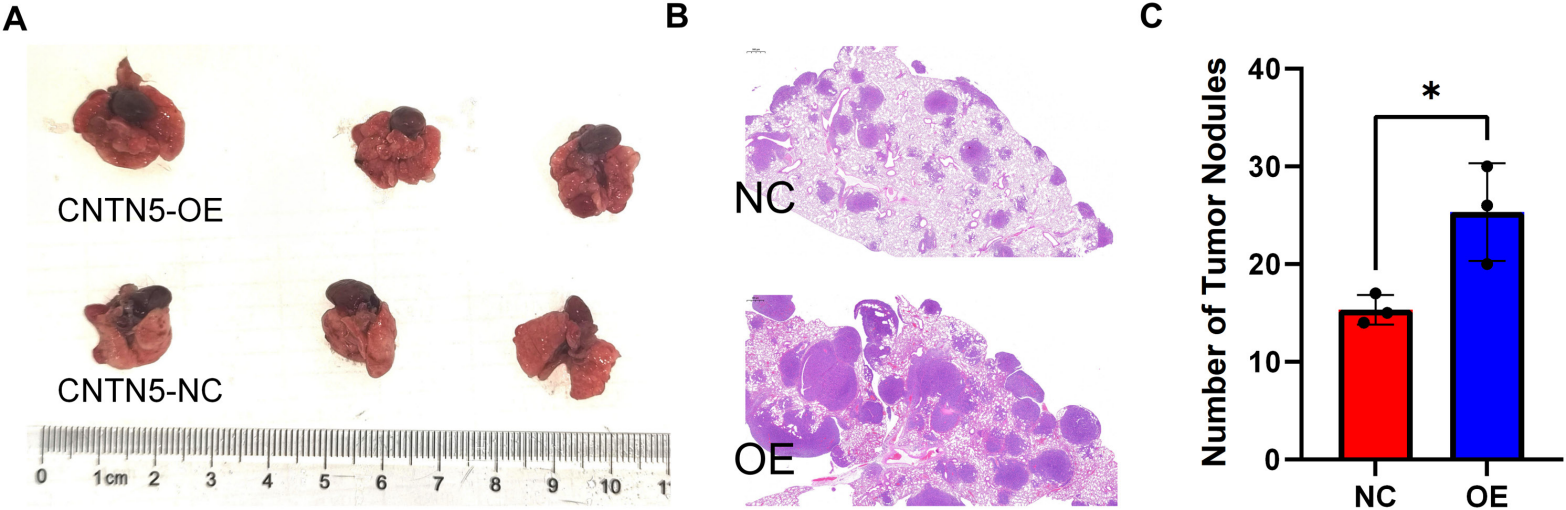
CNTN5 promotes NSCLC metastasis in vivo. (A) Metastatic nodules on the lung surface after necropsy. (B, C) Representative H&E‐stained lung sections showing metastatic lesions (*n* = 3). Quantitative data are presented as the mean ± SD. Comparisons between the control and CNTN5‐overexpression groups were performed using the two‐tailed unpaired Student's *t*‐test. Scale bar = 500 μm. *p < 0.05, **p < 0.01, ***p < 0.001.

To determine whether the pro‐metastatic effects observed in vitro could be recapitulated in vivo, a lung metastasis model was established by injecting H1975 cells stably overexpressing CNTN5 and control cells into nude mice. Histological examination using H&E staining revealed that mice injected with CNTN5‐overexpressing cells developed a significantly greater number of metastatic lung nodules, with larger lesion sizes, compared with control mice (Figure 3A–C). No significant difference in body weight changes was observed between the control and Cntn5‐overexpression groups. Consistently, the metastasis model using Lewis lung cancer cells also showed that Cntn5 overexpression increased lung metastatic colonization (Figure S4). These results demonstrate that CNTN5 enhances the metastatic potential of NSCLC cells in vivo.

### 3.4 Identification of Downstream Signaling Pathways Regulated by CNTN5

**FIGURE 4:**
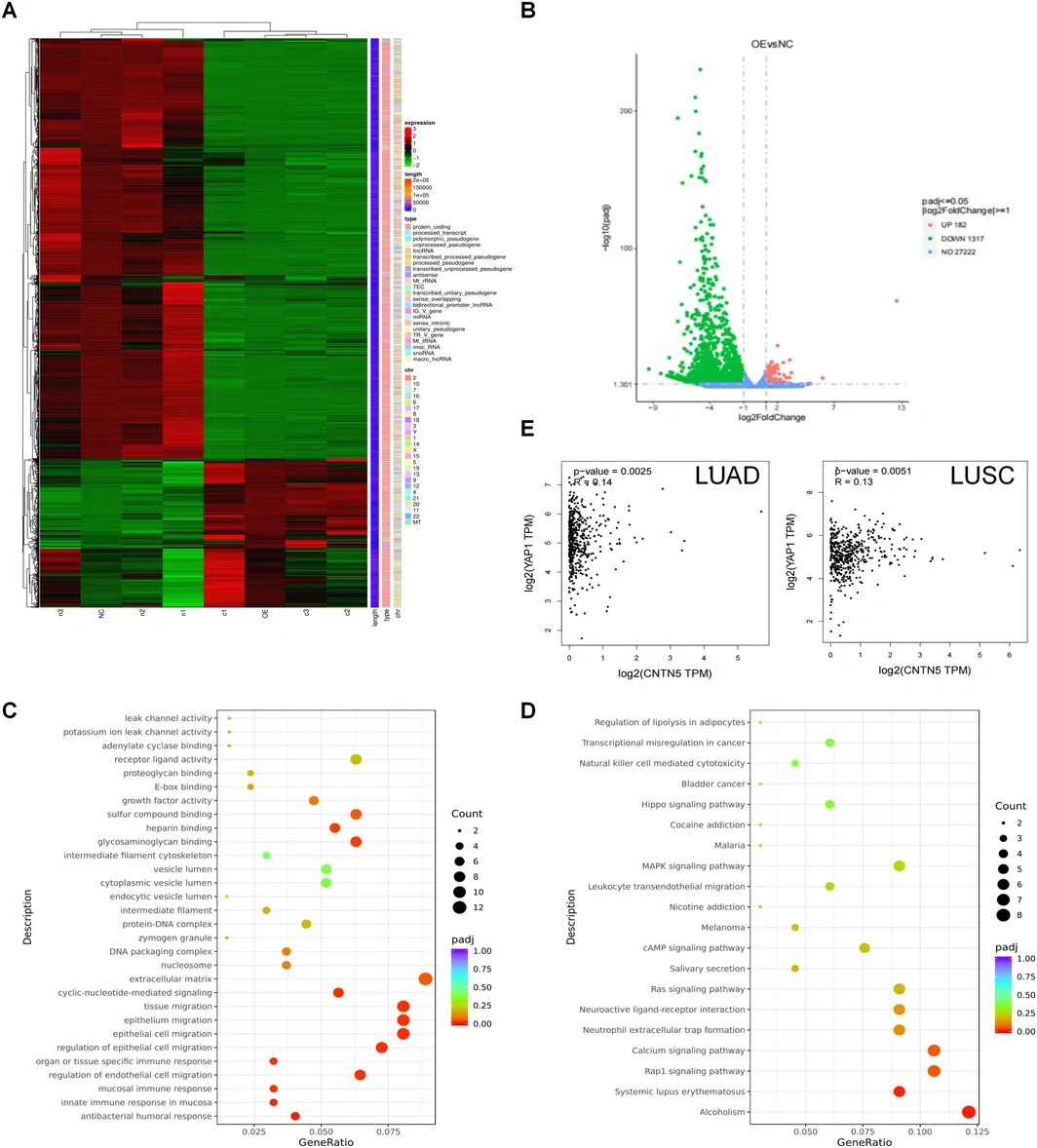
Identification of downstream signaling pathways regulated by CNTN5. (A, B) Heat maps and volcano plot of differentially expressed genes in CNTN5‐overexpressing H1299 cells. RNA sequencing was performed using three independent biological replicates per group. DEGs were defined by an adjusted *p*‐value < 0.05 and |log2 (fold change)| ≥ 1. (C, D) GO (C) and KEGG (D) analyses of CNTN5 co‐expression genes. (E) Correlations between CNTN5 expression and YAP1 expression.

To explore the molecular mechanisms underlying CNTN5‐mediated NSCLC metastasis, RNA sequencing was performed on CNTN5‐OE and control H1299 cells. A total of 1499 differentially expressed genes (DEGs) were identified (adjusted *p* < 0.05, |log2 (fold change)| ≥ 1), including 182 upregulated genes and 1317 downregulated genes (Figure 4A,B).

Gene ontology (GO) enrichment analysis revealed that genes involved in cell migration and immune‐related biological processes (BPs) were significantly enriched in CNTN5‐overexpressing cells (Figure 4C). KEGG pathway analysis further showed that cancer‐related signaling pathways, including the Hippo pathway, were significantly enriched in the CNTN5‐overexpression group (Figure 4D). Correlation analysis also showed that CNTN5 expression was positively correlated with YAP1 expression in NSCLC (Figure 4E). These results suggest that CNTN5 may promote NSCLC invasion and metastasis through regulation of the Hippo signaling pathway.

### 3.5 CNTN5 Promotes Migratory and Invasive Abilities Through YAP1

**FIGURE 5:**
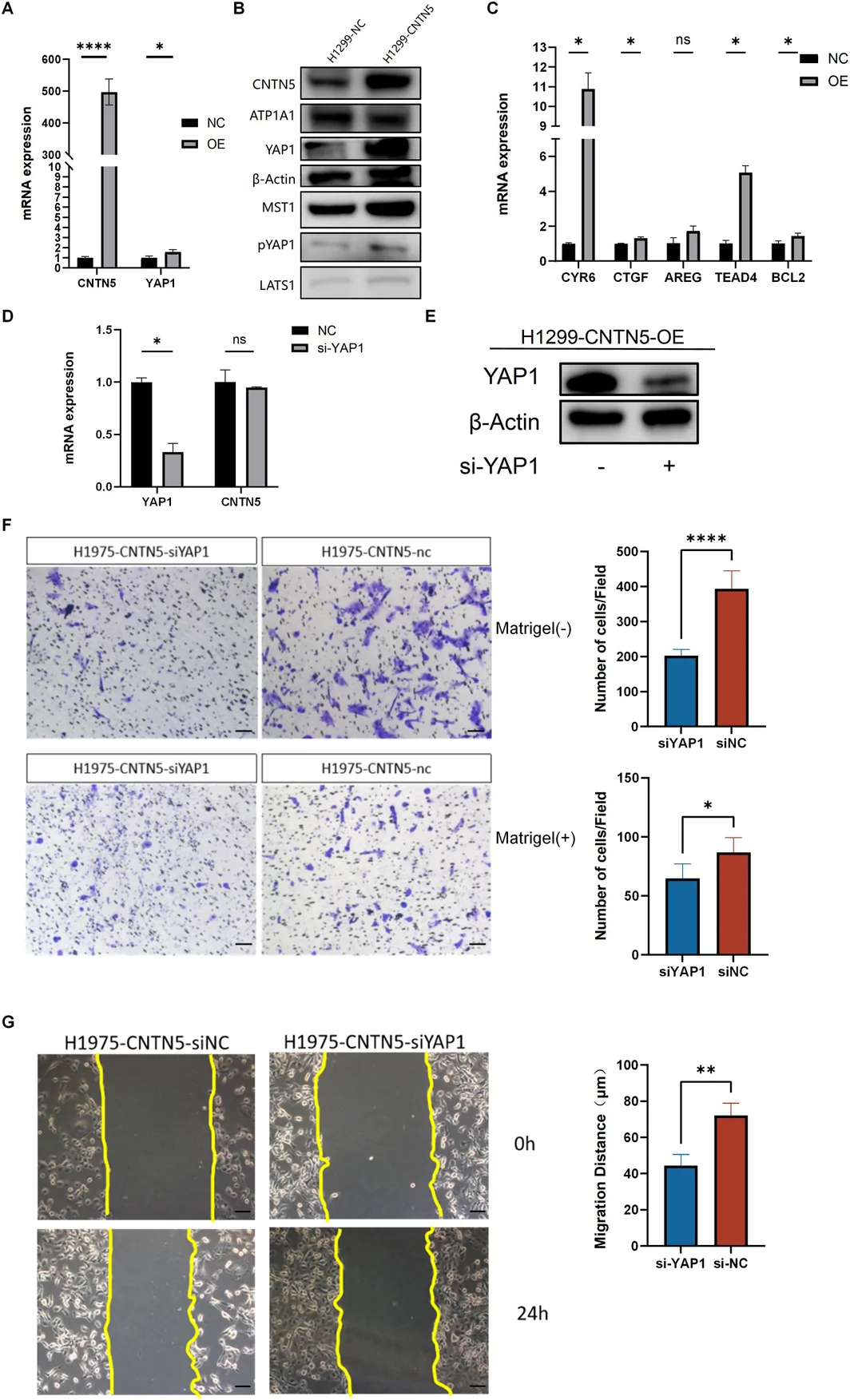
CNTN5 promoted migratory and invasive phenotypes through YAP1. (A) The effects of CNTN5 overexpression on the expression of YAP1 mRNA in H1299 cells, verified by RT‐qPCR. (B) Expression of YAP1 and Hippo pathway‐related proteins in CNTN5‐overexpressing and control H1299 cells was assessed by western blotting. (C) mRNA expression of YAP1 downstream target genes was measured by RT‐qPCR. (D, E) YAP1 expression in H1299 cells transfected with siYAP1 was detected by RT‐qPCR (D) and Western blotting (E). (F) The effect of YAP1 knockdown on the cell invasion and migration of CNTN5‐overexpressing H1975 cells, analyzed using Transwell assays. (G) The effect of YAP1 on the cell migration of CNTN5‐overexpressing H1975 cells, examined using the wound healing assay. Data are presented as the mean ± SD from at least three independent experiments (*n* ≥ 3). Comparisons were performed using the two‐tailed unpaired Student's *t*‐test. Scale bar = 100 μm. *p < 0.05, **p < 0.01, ***p < 0.001, ****p < 0.0001.

Based on transcriptomic analysis, we further focused on changes in YAP1, a key effector of the Hippo signaling pathway. Western blotting and RT‐qPCR analyses demonstrated that CNTN5 overexpression significantly increased YAP1 protein levels and mildly increased YAP1 mRNA levels, while Hippo pathway‐related proteins such as MST1/2 and phosphorylated YAP1 (p‐YAP1) also showed increasing trends (Figure 5A,B). Conversely, CNTN5 knockdown reduced YAP1‐related signaling activity compared with control cells, supporting the involvement of CNTN5 in YAP1 regulation (Figure S5). These results suggest that CNTN5 may regulate YAP1 activity through a noncanonical or context‐dependent mechanism, rather than simply suppressing the canonical Hippo kinase cascade.

To further evaluate YAP1 activity, we examined the expression of canonical YAP1 downstream target genes, including CYR61 and CTGF, which were markedly upregulated at the transcriptional level (Figure 5C). Subsequently, YAP1 was silenced using siRNA in CNTN5‐OE cells (Figure 5D,E). Transwell and wound healing assays were then performed to assess changes in cell migration and invasion (Figures 5F,G and S6). The results showed that YAP1 knockdown partially reversed the enhanced migratory and invasive abilities induced by CNTN5 overexpression. These findings supported YAP1 as an important downstream mediator of CNTN5‐induced migratory and invasive phenotypes.

### 3.6 CNTN5 Promotes YAP1 Nuclear Translocation via PTPN13

**FIGURE 6:**
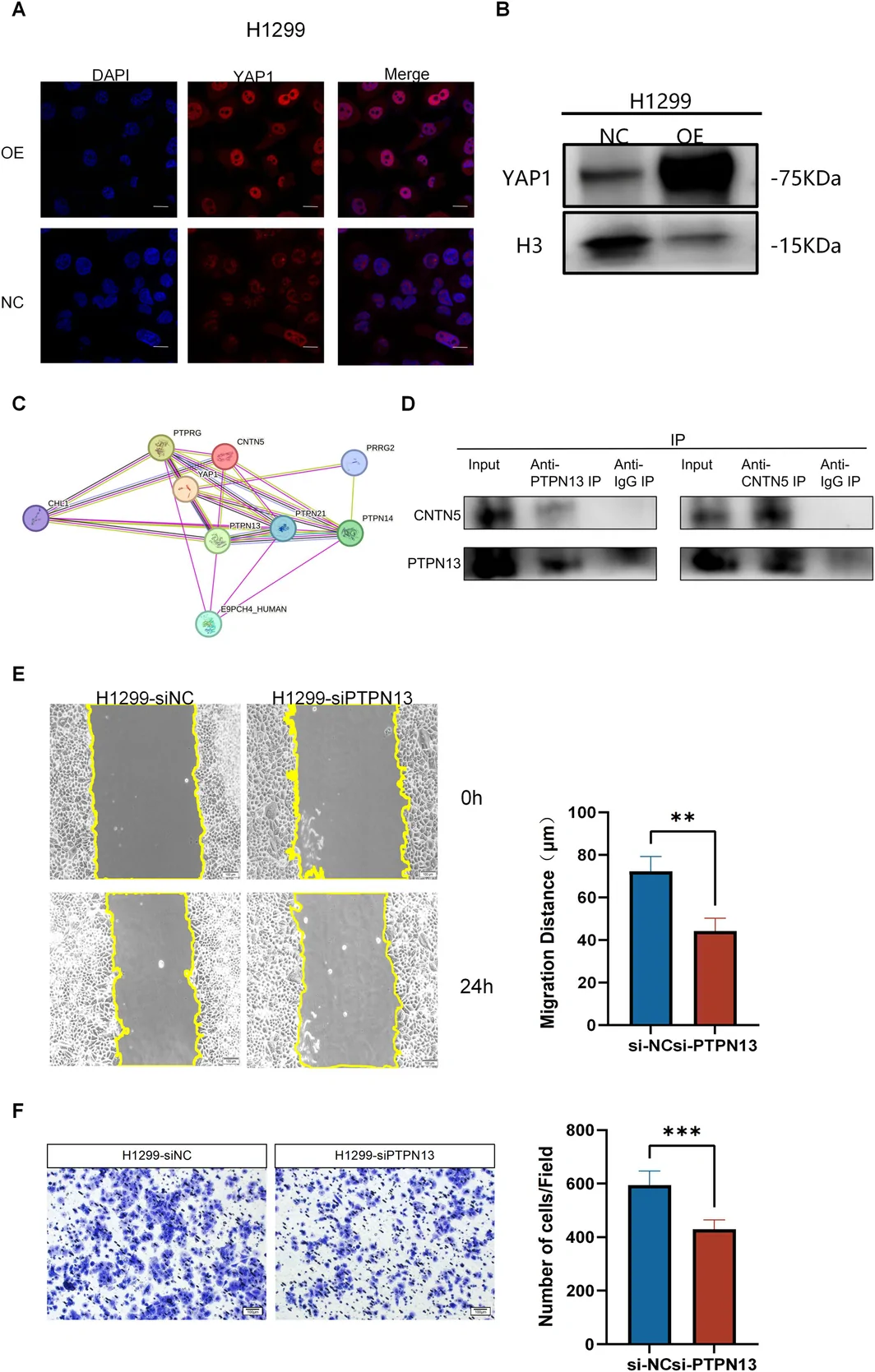
CNTN5 promoted YAP1 nuclear translocation via PTPN13. (A) Immunofluorescence staining of YAP1 nuclear localization after CNTN5 overexpression. Representative immunofluorescence images showing YAP1 staining (red) and nuclear counterstaining with DAPI (blue) in control cells and CNTN5‐overexpressing cells. (B) The effects of CNTN5 overexpression on the expression of nuclear YAP1 in H1299 cells, verified by western blotting after nuclear–cytoplasmic fractionation. (C) PPI network of CNTN5 co‐expressed proteins generated using STRING. Edges represent protein–protein associations rather than necessarily direct physical binding. Edge colors indicate different types of supporting evidence: cyan, curated database evidence; magenta, experimentally determined interactions; green, gene neighborhood; red, gene fusion; blue, gene co‐occurrence; yellow‐green, text mining; black, co‐expression; and purple, protein homology. (D) In H1299 cells, bidirectional IP confirmed that CNTN5 co‐precipitates with PTPN13. (E) The effect of PTPN13 knockdown on H1299 cell migration, investigated through the wound healing assay. (F) The effect of PTPN13 knockdown on H1299 cell invasion, analyzed using a Transwell assay. Quantitative data are presented as the mean ± SD from at least three independent experiments (*n* ≥ 3). Two‐group comparisons were performed using the two‐tailed unpaired Student's *t*‐test. Scale bar = 10 μm in immunofluorescence images and 100 μm in wound healing and Transwell images. *p < 0.05, **p < 0.01, ***p < 0.001.

Given that YAP1 transcriptional activity depends on its nuclear localization, we examined its subcellular distribution. Immunofluorescence staining and nuclear–cytoplasmic fractionation assays consistently demonstrated that CNTN5 overexpression markedly promoted nuclear accumulation of YAP1 (Figure 6A,B). Moreover, YAP1 knockdown reduced nuclear YAP1 levels (Figure S7), suggesting that nuclear YAP1 accumulation is functionally involved in CNTN5‐mediated malignant behavior.

To investigate the molecular basis by which CNTN5 regulates Hippo–YAP1 signaling, STRING database analysis and bioinformatics screening identified that YAP1 was connected to CNTN5 through the protein tyrosine phosphatase PTPN13, suggesting that PTPN13 may function as a potential key mediator (Figure 6C). In addition, correlation analysis showed a positive association between CNTN5 and PTPN13 expression (Figure S8A). Co‐immunoprecipitation (Co‐IP) assays further supported a physical interaction between CNTN5 and PTPN13 (Figure 6D).

To further evaluate the functional relevance of PTPN13, we silenced PTPN13 using siRNA. Western blotting showed that PTPN13 silencing reduced CNTN5 expression and decreased nuclear YAP1 levels (Figure S8B). Functionally, PTPN13 knockdown significantly suppressed the migratory and invasive abilities of NSCLC cells (Figure 6E,F). Collectively, these findings suggest that CNTN5 may interact with PTPN13 to modulate Hippo–YAP signaling, leading to increased nuclear localization and transcriptional activity of YAP1, thereby contributing to the migratory and invasive phenotype of NSCLC cells.

### 3.7 Discussion

In this study, we systematically evaluated the biological role of CNTN5 in NSCLC progression. By integrating public databases and clinical specimens, we found that CNTN5 is upregulated in NSCLC and highly expressed in metastatic tumor tissues. Functionally, CNTN5 increased tumor cell migration and invasion and enhanced metastatic burden in vivo. Transcriptomic profiling together with protein level validation further suggested that CNTN5 promotes a metastatic phenotype by facilitating nuclear translocation of YAP1, the core transcriptional coactivator of the Hippo pathway.

CNTN5 is a member of the Contactin family, a group of glycosylphosphatidylinositol (GPI)‐anchored immunoglobulin superfamily cell adhesion molecules that play essential roles in axon guidance and synapse formation [5, 20]. Genetic studies have also suggested that variants in several family members are associated with neurodevelopmental abnormalities. In recent years, increasing evidence has indicated that the functions of the CNTN family extend beyond the nervous system, with emerging roles in cancer progression. For example, CNTN1 exhibited pro‐invasive and pro‐metastatic behavior in multiple solid tumors and has been linked to epithelial‐mesenchymal transition [21, 22]. CNTN2 is upregulated in high‐grade glioma and interacts with RACK1, thereby activating the RTK/Ras/MAPK signaling cascade to promote proliferation and suppress differentiation [23]. Recent work further demonstrated a direct role of the CNTN family in tumor immune evasion: CNTN4 engages amyloid precursor protein (APP) on T cells, disrupts tumor cell‐T cell conjugation, and suppresses TCR signaling; blockade of the CNTN4‐APP axis can enhance antitumor T‐cell responses, suggesting a potential therapeutic strategy to overcome resistance to immunotherapy [24]. In addition, CNTN6 has been observed among recurrent hotspot genes in the landscape of HBV integration events in HBV‐related hepatocellular carcinoma [25]. Our work extended CNTN5 research into oncology. Importantly, increasing evidence suggested that other contactin family proteins contribute to tumor progression [21, 24, 26]. Although CNTN5 has been intermittently associated with tumor‐related features in prior studies, its functional contribution and mechanistic basis in lung cancer have remained unclear [13]. Here, we provide evidence that CNTN5 participates in cancer cell invasion and metastasis, thereby expanding the biological spectrum of contactin family members and offering a new molecular perspective on NSCLC aggressiveness.

The Hippo pathway is a central signaling network that coordinates cell adhesion, proliferation, and cytoskeletal dynamics to regulate migratory and invasive behavior [27, 28]. Aberrant activation of YAP/TAZ transcriptional programs has been associated with metastasis, therapeutic resistance, and adverse outcomes in multiple cancers, including NSCLC [29, 30, 31]. In our study, CNTN5 overexpression increased nuclear YAP1 accumulation and induced transcription of canonical downstream target genes. These findings indicated that CNTN5‐induced metastatic phenotypes may be mediated by YAP1. We observed higher total YAP1 abundance and nuclear localization under CNTN5 overexpression, while phosphorylation signals at upstream Hippo components also increased. This pattern suggested that CNTN5 may enhance YAP transcriptional activity primarily by improving YAP stability and nuclear shuttling efficiency, rather than solely by suppressing the canonical LATS kinase cascade. Notably, Hippo signaling is highly dynamic and involves feedback regulation and pathway crosstalk [32, 33]. Therefore, whether CNTN5 regulates YAP1 through additional pathways warrants further investigation.

To explore how CNTN5 regulates YAP1, we identified and experimentally validated PTPN13 as a potential mediator. PTPN13 is a nonreceptor protein tyrosine phosphatase [34]. It has been implicated in the regulation of cell growth, apoptosis, and tumorigenesis in cancer [35, 36, 37]. In this study, STRING‐based protein association analysis and correlation analysis supported a relationship between CNTN5 and PTPN13, while Co‐IP assays further supported an interaction between CNTN5 and PTPN13. Functionally, PTPN13 knockdown reduced nuclear YAP1 levels and suppressed migration and invasion. We propose that the interaction between CNTN5 and PTPN13 may alter the subcellular localization or enzymatic activity of PTPN13. This change may indirectly affect YAP1 homeostasis and nuclear translocation, leading to its increased nuclear accumulation. Future studies should further define the composition and function of this complex in more detail, including whether PTPN13 directly dephosphorylates YAP or a key upstream YAP regulatory component.

This study has several limitations. First, although our animal experiments support the pro‐metastatic role of CNTN5, larger clinical cohorts are still required. Second, as a GPI‐anchored surface protein, upstream mechanisms controlling expression of CNTN5 remain unclear. Signals from the tumor microenvironment may contribute to CNTN5 dysregulation and should be investigated in future work.

## 4 Conclusion

Overall, CNTN5 promotes metastatic traits in NSCLC and may do so via the Hippo‐YAP signaling axis, with PTPN13 potentially involved in this regulatory process, supporting its potential value as a prognostic biomarker and therapeutic target.

## Author Contributions

Conceptualization: Jiarui Wang. Methodology: Jiarui Wang, Mengtao Wang, Yanze Yin, Ao Zeng. Investigation: Jiarui Wang, Mengtao Wang, Keyi Chen, Zhilong Xu. Data curation: Jiarui Wang. Formal analysis: Jiarui Wang. Writing – original draft: Jiarui Wang. Writing – review and editing: Jiarui Wang, Jie Dai. Supervision: Jie Dai.

## Ethics Statement

Approval for the collection and utilization of clinical samples was granted by the Ethics Committee of Shanghai Pulmonary Hospital (Ethical approval number: K23–228). Informed consent was obtained from all subjects and/or their legal guardian(s). All procedures involving human specimens were conducted in accordance with the Declaration of Helsinki. All animal experiments were approved by the Committee on the Ethics of Animal Experiments of Shanghai Pulmonary Hospital (ethical approval number: K21‐097Y) and were conducted in accordance with the institutional guidelines for the care and use of laboratory animals.

## Conflicts of Interest

The authors declare no conflicts of interest.

## Supporting information


**Figure S1:** CNTN5 is upregulated in NSCLC cell lines. (A) RT‐qPCR analysis of CNTN5 mRNA expression in normal lung epithelial cell line and NSCLC cell lines. (B) Western blotting of CNTN5 protein expression in normal lung epithelial cell line and NSCLC cell lines.
**Figure S2:** CNTN5 overexpression did not promote apoptosis in H2199 cells. Cell apoptosis was detected by Annexin V‐FITC/PI double staining followed by flow cytometry analysis. (A) Representative scatter plots of flow cytometry. (B) Quantitative analysis of the percentages of early and late apoptotic cells. nd: Not different. Quantitative data are presented as the mean ± SD from at least three independent experiments (n ≥ 3). Two‐group comparisons were performed using the two‐tailed unpaired Student's t‐test.
**Figure S3:** Knockdown of CNTN5 inhibits the migration and invasion of NSCLC cells. (A) Representative images of the wound healing (left panel). Quantitative analysis of cell migration rate, calculated based on the wound area. (Right panel) (B) Representative images of the Transwell (left panel) and quantitative analysis of cell migration or invasion rate. Quantitative data are presented as the mean ± SD from at least three independent experiments (n ≥ 3). Two‐group comparisons were performed using the two‐tailed unpaired Student's t‐test. Scale bar = 100 μm. *p < 0.05, **p < 0.01, ***p < 0.001, ****p < 0.0001.
**Figure S4:** The effect of Cntn5 on the metastasis of Lewis lung cancer was investigated through in vivo experiments. (A) Metastatic nodules on the surface of the lung tissues of mice after dissection and removal. (B, C) Representative H&E‐stained lung sections and quantification of metastatic nodules. (D) Body weight changes during the 14‐day experimental period. Quantitative data are presented as the mean ± SD from at least three independent experiments (n ≥ 3). Two‐group comparisons were performed using the two‐tailed unpaired Student's t‐test. CCK‐8 data were analyzed using two‐way ANOVA. *p < 0.05, **p < 0.01, ***p < 0.001, ****p < 0.0001.
**Figure S5:** The effects of CNTN5 knockdown on the expression of Hippo signaling pathway in H1299 cells, verified by Western blotting.
**Figure S6:** YAP1 knockdown attenuated CNTN5‐induced migration and invasion in H1299 cells. (A) Transwell assays showed the effect of YAP1 knockdown on the invasion and migration of CNTN5‐overexpressing H1299 cells. (B) Wound healing assay showed the effect of YAP1 knockdown on cell migration. Scale bar = 100 μm. Quantitative data are presented as the mean ± SD from at least three independent experiments (n ≥ 3). Two‐group comparisons were performed using the two‐tailed unpaired Student's t‐test. *p < 0.05, ****p < 0.0001.
**Figure S7:** (A) Immunofluorescence staining of YAP1 nuclear localization after YAP1 knockdown by siRNA. Representative immunofluorescence images showing YAP1 staining (red) and nuclear counterstaining with DAPI (blue) in YAP1 knockdown cells and CNTN5‐overexpressing cells. (B) The effects of YAP1 knockdown on the expression of nuclear YAP1 in H1299 cells, verified by Western blotting after nuclear–cytoplasmic fractionation. Scale bar = 10 μm.
**Figure S8:** PTPN13 is positively associated with CNTN5 and regulates nuclear YAP1 expression. (A) Correlations between CNTN5 expression and PTPN13 expression in NSCLC. (B) Western blotting showed the effects of PTPN13 knockdown on CNTN5 expression and nuclear YAP1 levels in H1299 cells. β‐actin, ATP1A1, and H3 were used as loading controls for total protein, membrane protein and nuclear protein, respectively.

**Table S1:** Primer sequences used in this study.

## Data Availability

The datasets generated or analyzed during the current study are available from the corresponding author upon reasonable request.
