# Supplementary material for: CNTN5 Promotes Invasion and Metastasis in Non‐Small Cell Lung Cancer by Regulating YAP1 Nuclear Translocation
Source: J Gene Med. 2026 Sep 21;28(9):e70112. doi: 10.1002/jgm.70112 (PMC13591293; doi:10.1002/jgm.70112)
Supplement: Supplementary file 1 [file JGM-28-e70112-s001.pdf]

Fig. S1.

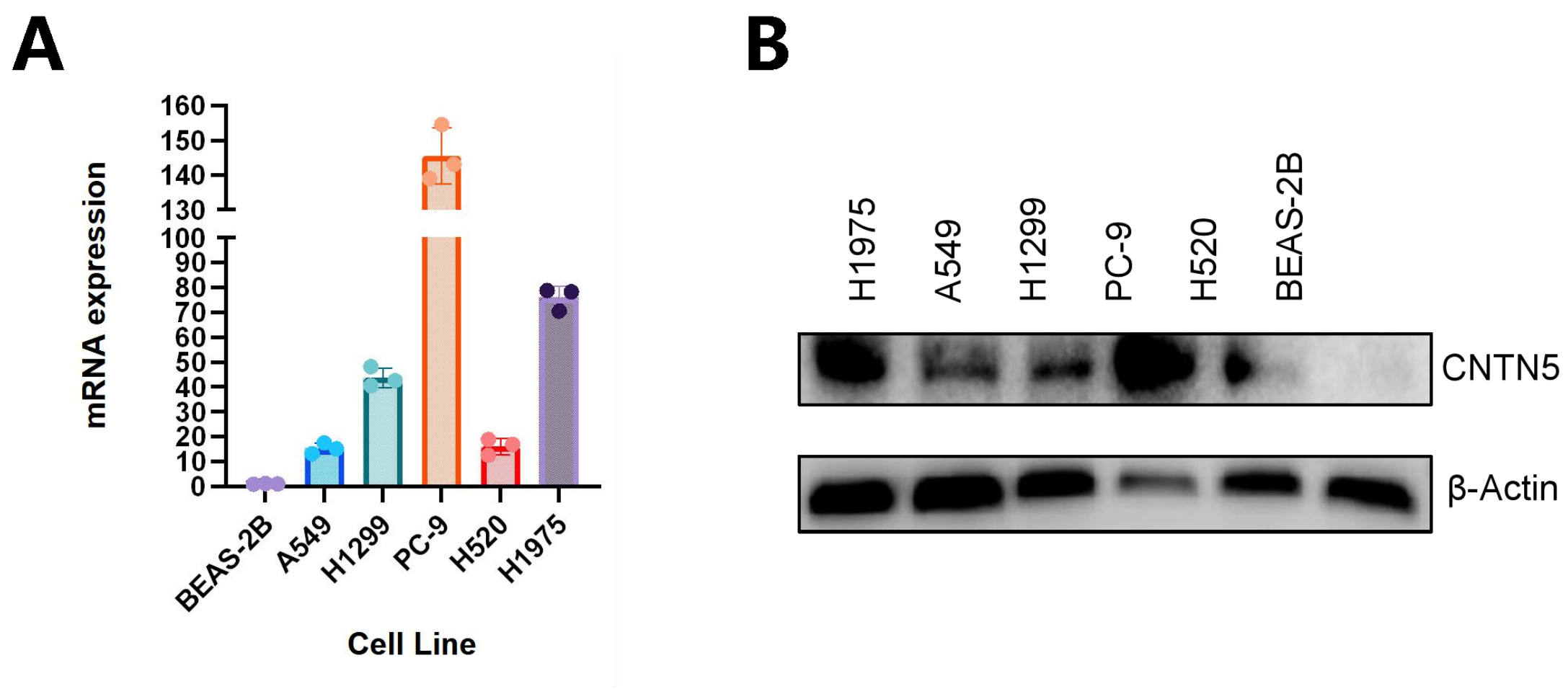

Fig. S1. CNTN5 is upregulated in NSCLC cell lines. (A) RT-qPCR analysis of CNTN5 mRNA expression in normal lung epithelial cell line and NSCLC cell lines. (B) Western blotting of CNTN5 protein expression in normal lung epithelial cell line and NSCLC cell lines.

Fig. S2.

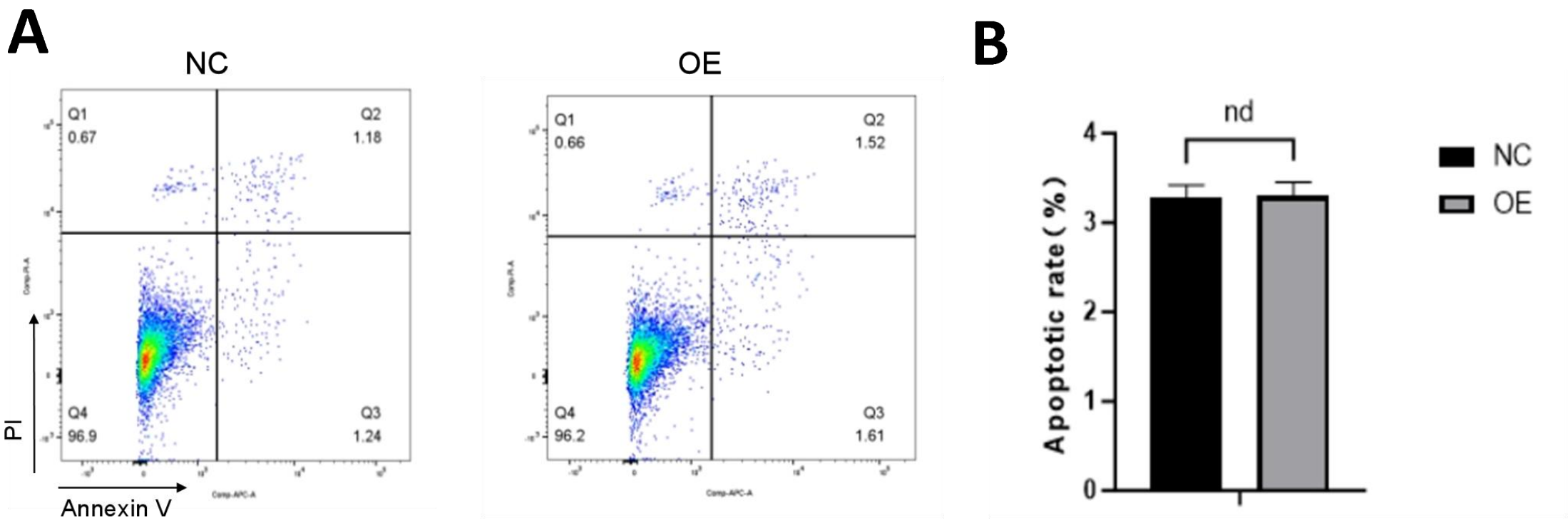

Fig. S2. CNTN5 overexpression did not promote apoptosis in H2199 cells. Cell apoptosis was detected by Annexin V-FITC/PI double staining followed by flow cytometry analysis. (A) Representative scatter plots of flow cytometry. (B) Quantitative analysis of the percentages of early and late apoptotic cells. nd: Not Different. Quantitative data are presented as the mean ± SD from at least three independent experiments (n ≥ 3) . Two-group comparisons were performed using the two-tailed unpaired Student's t-test.

Fig. S3.

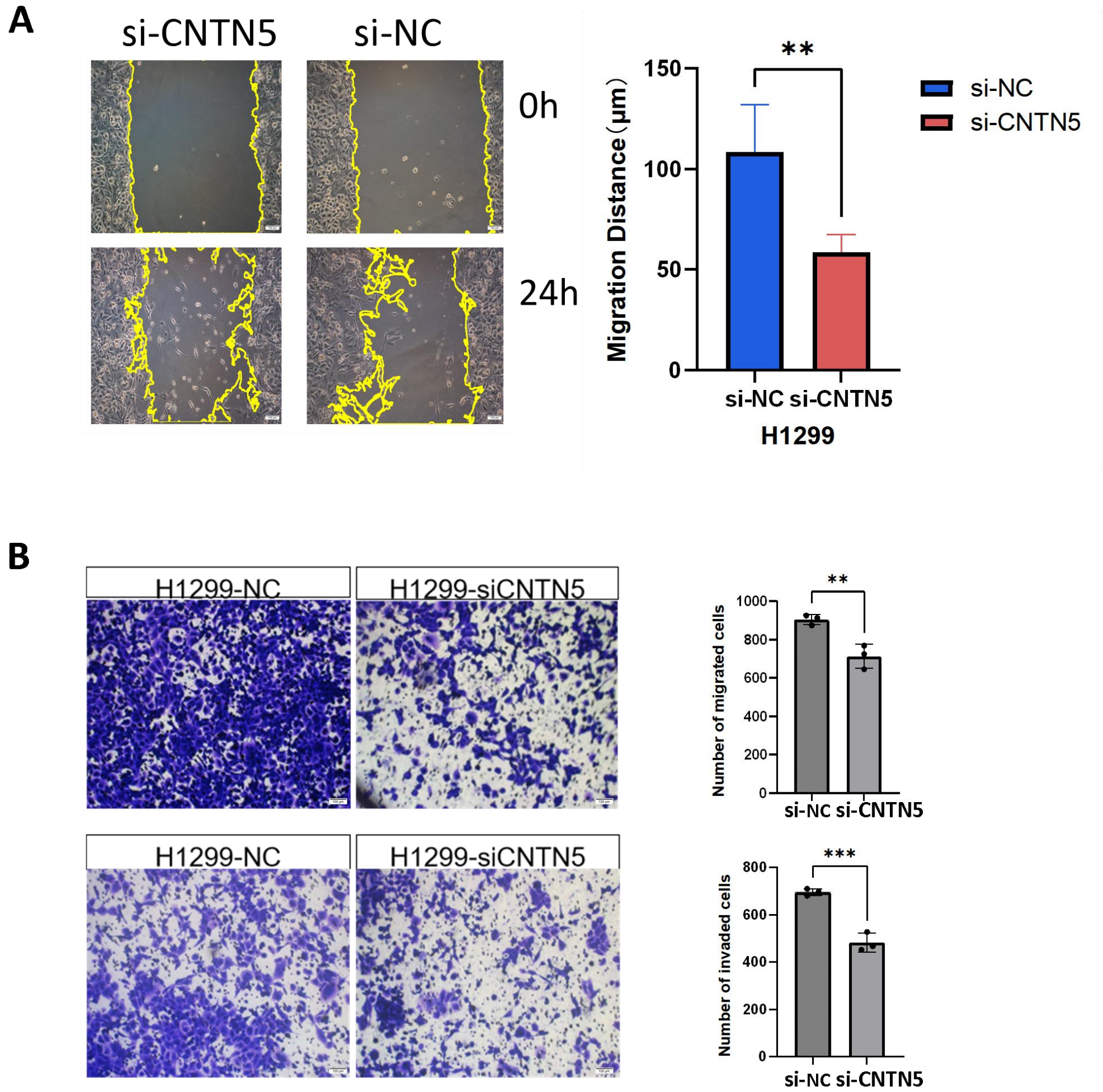

Fig. S3. Knockdown of CNTN5 inhibits the migration and invasion of NSCLC cells. (A) Representative images of the wound healing (left panel). Quantitative analysis of cell migration rate, calculated based on the wound area.(Right panel) (B) Representative images of the Transwell (left panel) and uantitative analysis of cell migration or invasion rate. Quantitative data are presented as the mean ± SD from at least three independent experiments (n ≥ 3) . Two-group comparisons were performed using the two-tailed unpaired Student's t-test. Scale bar = 100 μm. *P < 0.05, **P < 0.01, ***P < 0.001, ****P < 0.0001.

Fig. S4.

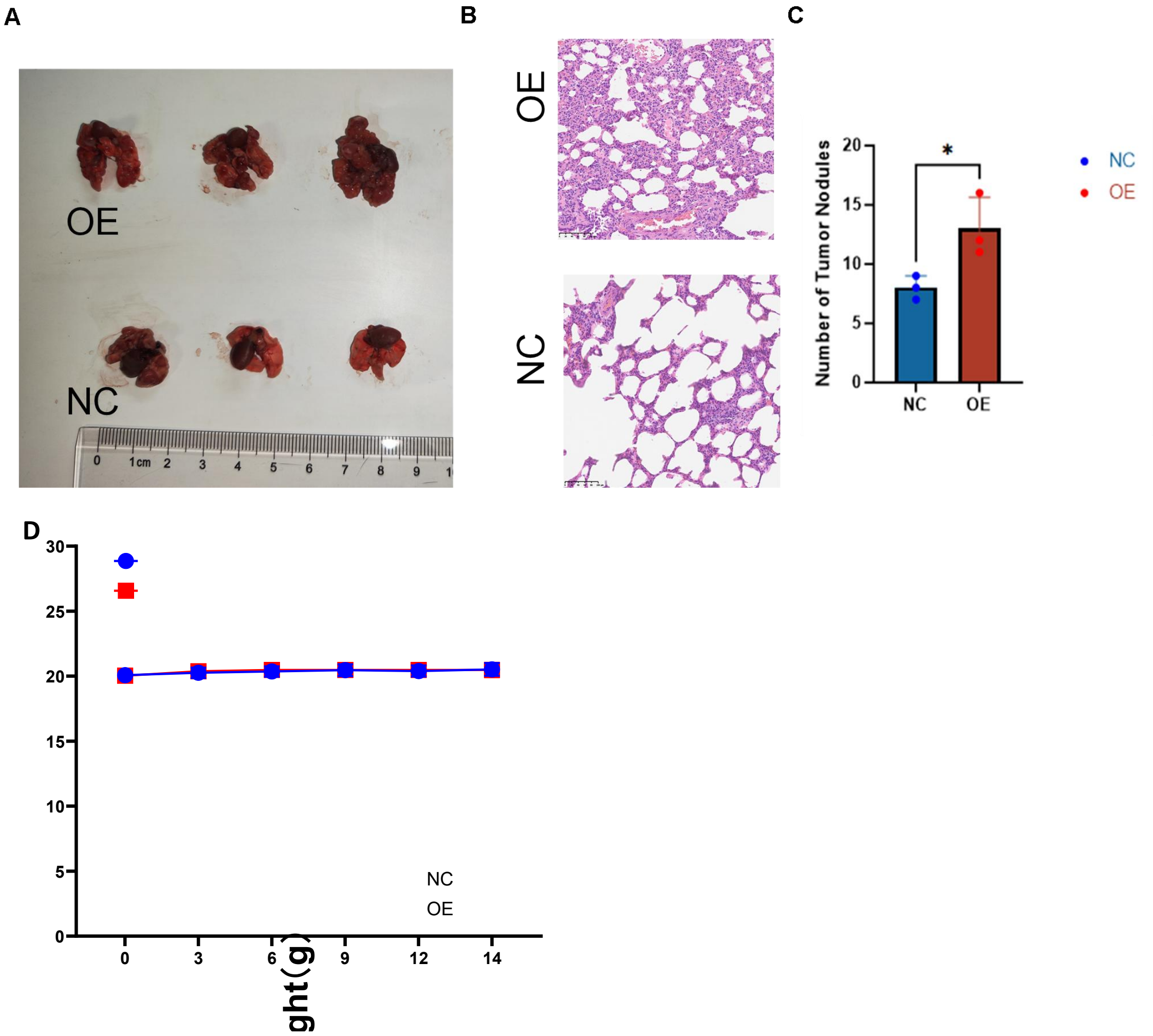

Fig. S4. The effect of Cntn5 on the metastasis of Lewis lung cancer was investigated through in vivo experiments. (A) Metastatic nodules on the surface of the lung tissues of mice after dissection and removal. (B&C) Representative H&E-stained lung sections and quantification of metastatic nodules. (D) Body weight changes during the 14-day experimental period. Quantitative data are presented as the mean ± SD from at least three independent experiments (n ≥ 3). Two-group comparisons were performed using the two-tailed unpaired Student's t-test. CCK-8 data were analyzed using two-way ANOVA. *P < 0.05, **P < 0.01, ***P < 0.001, ****P < 0.0001.

Fig. S5.

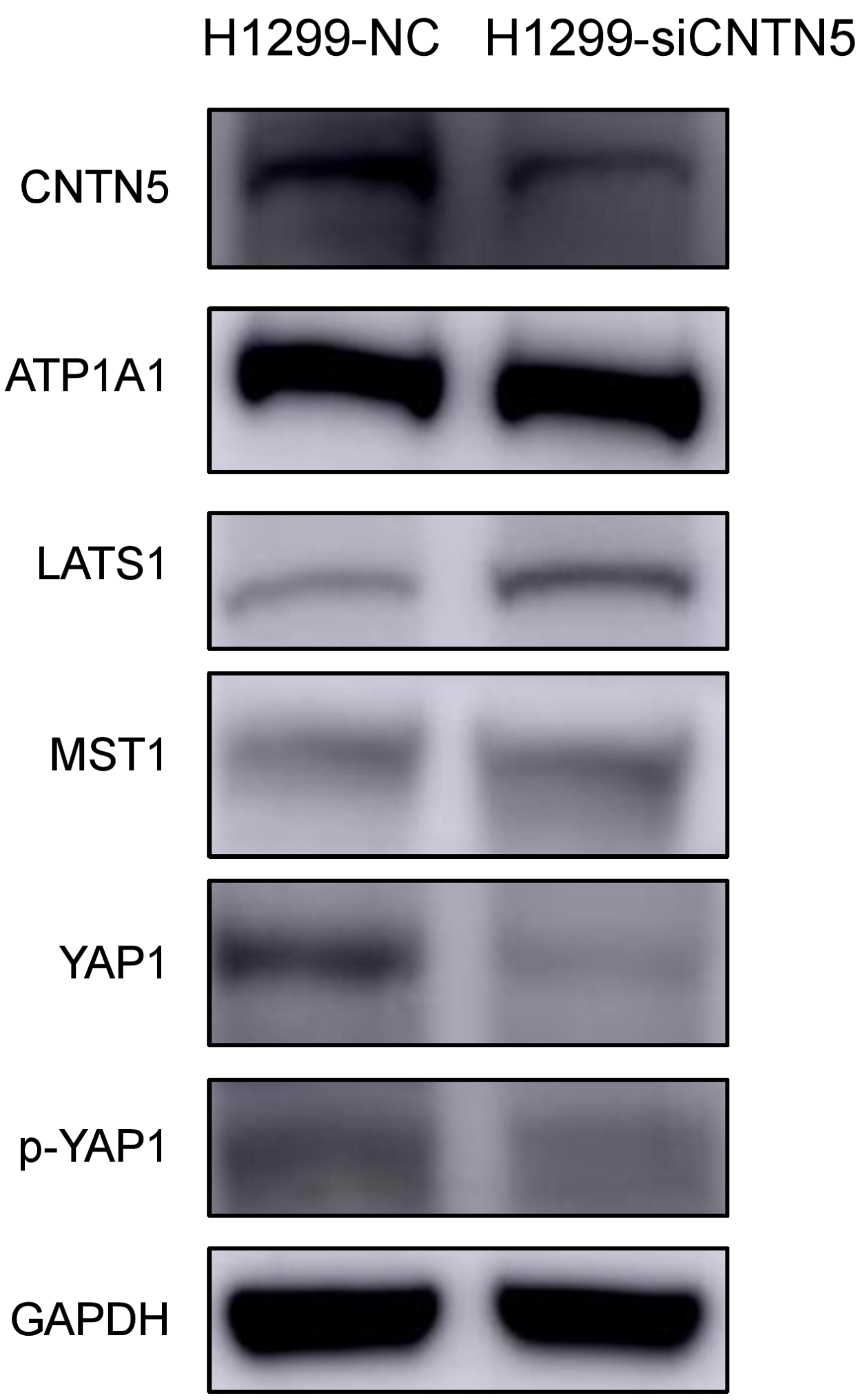

Fig. S5. The effects of CNTN5 knockdown on the expression of Hippo-signaling pathway in H1299 cells, verified by Western blotting.

Fig. S6.

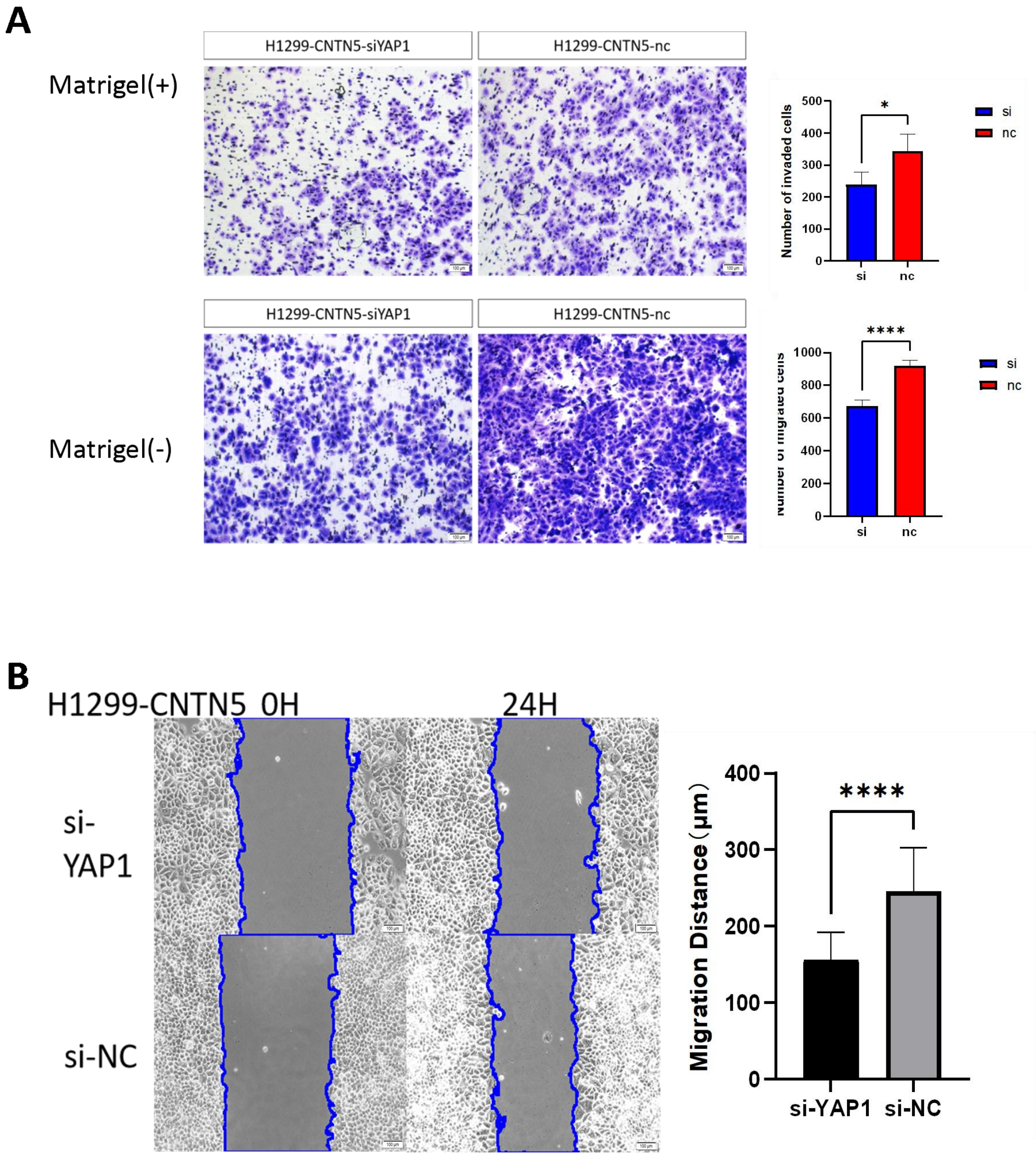

Fig. S6. YAP1 knockdown attenuated CNTN5-induced migration and invasion in H1299 cells. (A) Transwell assays showed the effect of YAP1 knockdown on the invasion and migration of CNTN5-overexpressing H1299 cells. (B) Wound-healing assay showed the effect of YAP1 knockdown on cell migration. Scale bar = 100 μm. Quantitative data are presented as the mean ± SD from at least three independent experiments (n ≥ 3). Two-group comparisons were performed using the two-tailed unpaired Student's t-test. *P < 0.05, ****P < 0.0001.

Fig. S7.

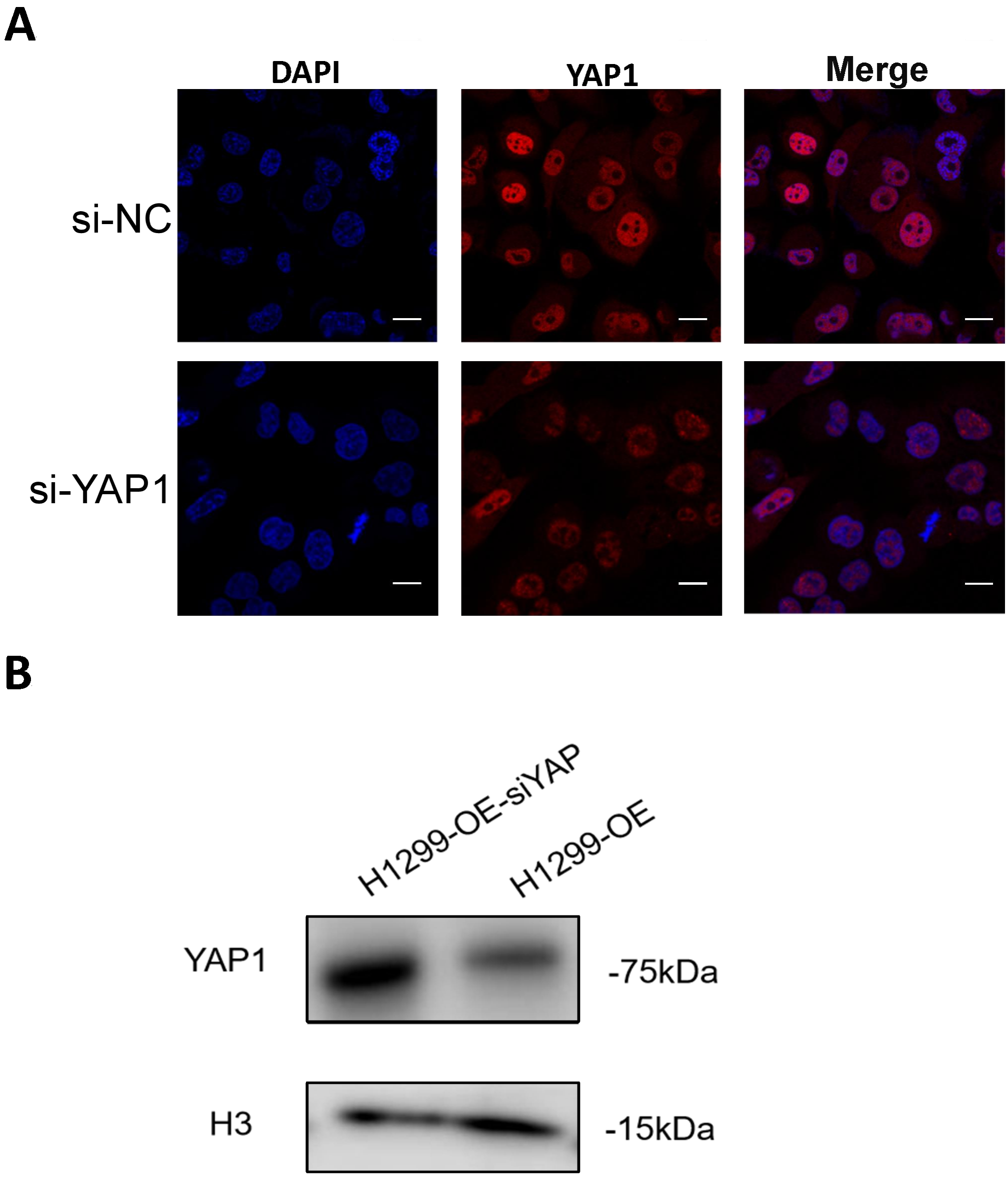

Fig. S7. (A) Immunofluorescence staining of YAP1 nuclear localization after YAP1 knockdown by siRNA. Representative immunofluorescence images showing YAP1 staining (red) and nuclear counterstaining with DAPI (blue) in YAP1 knockdown cells and CNTN5-overexpressing cells. (B) The effects of YAP1 knockdown on the expression of nuclear YAP1 in H1299 cells, verified by Western blotting after nuclear – cytoplasmic fractionation. Scale bar = 10 µm.

Fig. S8.

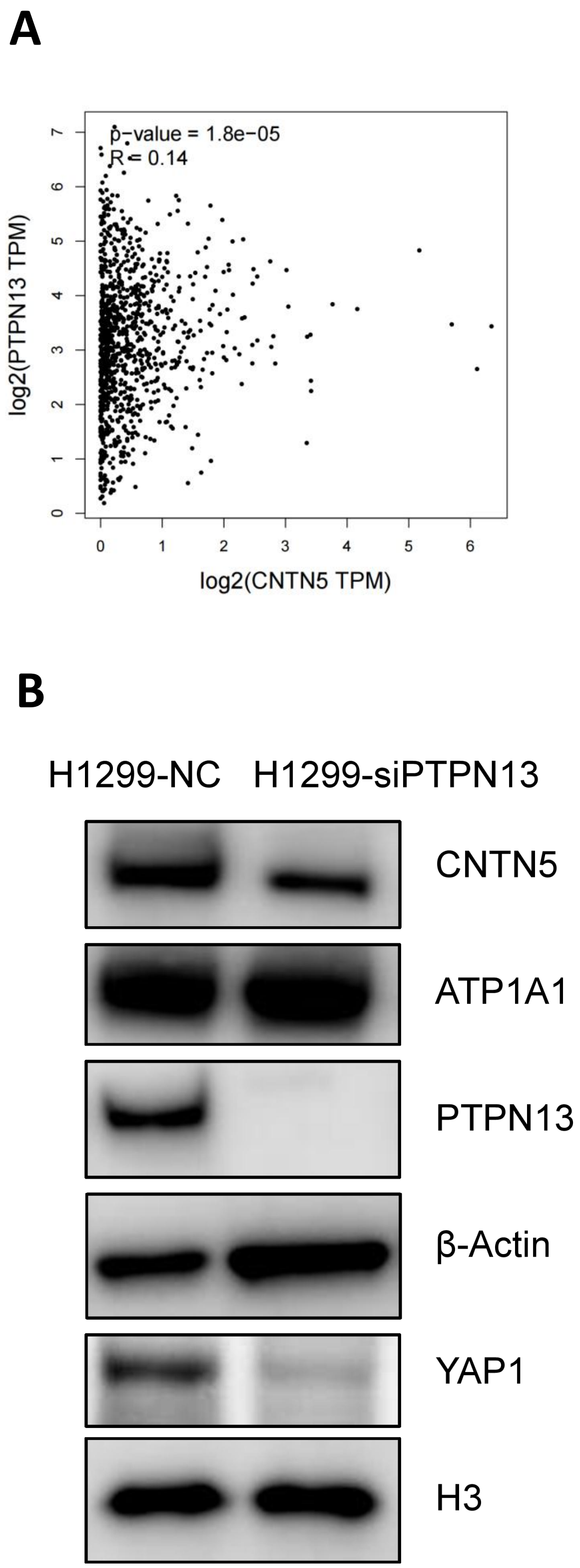

Fig. S8. PTPN13 is positively associated with CNTN5 and regulates nuclear YAP1 expression. (A) Correlations between CNTN5 expression and PTPN13 expression in NSCLC. (B) Western blotting showed the effects of PTPN13 knockdown on CNTN5 expression and nuclear YAP1 levels in H1299 cells. β-actin, ATP1A1 and H3 were used as loading controls for total protein, membrane protein and nuclear protein, respectively.
